# Treatment of Choroidal Metastasis from Epidermal Growth Factor Mutant Non-Small Cell Lung Cancer with First-line Osimertinib Therapy

**DOI:** 10.18502/jovr.v17i1.10178

**Published:** 2022-01-21

**Authors:** Anderson N. Vu, Urmi V. Mehta, Paul Israelsen, Sai-Hong Ignatius Ou, Andrew W. Browne

**Affiliations:** ^1^Gavin Herbert Eye Institute, Department of Ophthalmology, University of California Irvine, Irvine, CA, USA; ^2^Western University of Health Sciences, Pomona, CA, USA; ^3^Chao Family Comprehensive Cancer Center, Division of Hematology-Medical Oncology, Department of Internal Medicine, University of California Irvine, Orange, CA, USA; ^4^School of Medicine, University of California Irvine, Irvine, CA, USA; ^5^Institute for Clinical and Translational Sciences, University of California Irvine, Irvine, CA, USA; ^6^Department of Biomedical Engineering, Henry Samueli School of Engineering, University of California Irvine, Irvine, CA, USA

**Keywords:** Carcinoma, Choroidal Neoplasm, Multimodal Imaging, Neoplasm Metastasis, Non-small Cell Lung, Osimertinib

## Abstract

**Purpose:**

To illustrate the regression of a metastatic lesion through ophthalmic imaging and correlating findings with standard chest imaging and treatment with osimertinib, an oral chemotherapy agent specific to Epidermal Growth Factor Receptor + Non-small Cell Lung Cancer (EGFR+ NSCLC).

**Case Report:**

A 63-year-old Asian male presented to ophthalmology with a complaint of left blurry vision. Initial ophthalmic exam revealed a choroidal lesion and imaging results highlighted a spiculated lung mass with brain and bony metastases. Osimertinib was chosen for its specificity and ability to cross the blood–brain barrier. Follow-up ophthalmic and radiographic imaging were repeated over the course of treatment.

**Conclusion:**

After the initiation of osimertinib, ophthalmic and computed tomography imaging highlighted the regression of the ocular metastatic disease and primary malignancy, respectively.

Osimertinib is an effective first-line treatment of EGFR+ NSCLC and corresponding metastatic sites. Additionally, ophthalmic imaging can be used to monitor general response to chemotherapy agents when ocular metastasis is identified.

##  INTRODUCTION

The choroidal vasculature underlying the retina is the most common site for intraocular metastasis.^[1]^ Visual disturbances may sometimes be the first presenting sign of metastatic disease to the eye. The most common malignancies to metastasize to the choroid are breast, lung, and gastrointestinal tract.^[1]^ Once malignancy is suspected in the eye, prompt characterization is needed to determine therapy targeting the primary lesion and its dissemination. Osimertinib is an irreversible third-generation Epidermal Growth Factor Receptor Tyrosine Kinase Inhibitor (EGFR-TKI) that targets EGFR-TKI sensitizing and T790M resistance mutations and is the standard of care as first-line treatment of Epidermal Growth Factor Receptor + Non-small Cell Lung Cancer (EGFR+ NSCLC).^[2,3,4]^ We present a case of choroidal metastasis from a primary pulmonary malignancy with full characterization and follow-up using ophthalmic imaging as a clinical endpoint for treatment response to osimertinib.

##  CASE REPORT

A 63-year-old Asian male presented to ophthalmology clinic with complaint of central blurry vision of the left eye. Past medical history revealed well-controlled type II diabetes mellitus and no past ocular history. Best-corrected visual acuity in both eyes was 20/20, however, the patient reported blurry vision in the left eye. Pupillary responses, confrontational visual field testing, and intraocular pressure were normal.

**Figure 1:**
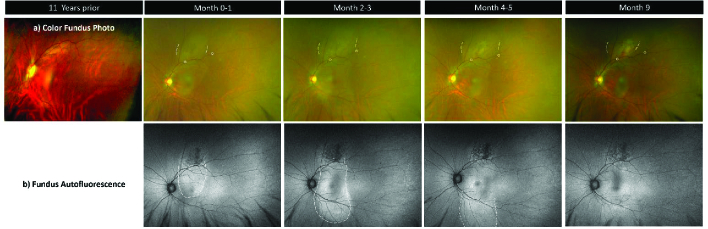
(a) Color fundus photography showing lateral boundaries (dotted lines) of tumor above fovea. White circles are the same bifurcation of vessels in each image. (b) Autofluorescence showing speckled pattern and extent of subretinal fluid (dotted lines).

**Figure 2:**
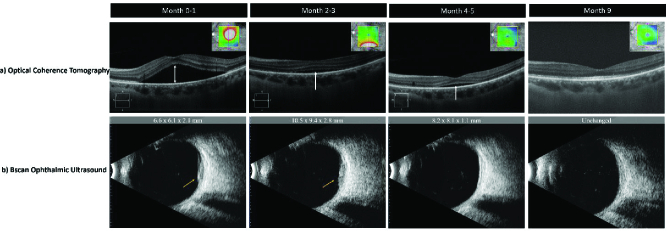
(a) Optical coherence tomography showing resolving subretinal fluid and flattening of retina. (b) Bscan ophthalmic ultrasound showing regression of metastatic lesion.

**Figure 3:**
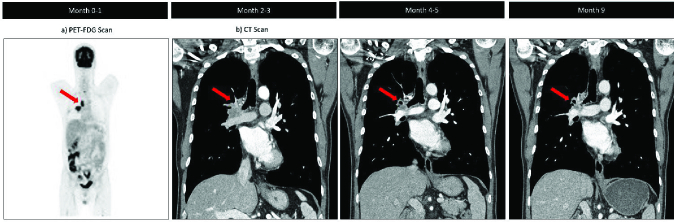
Positron emission and computed tomography showing primary epidermal growth factor receptor + non-small cell lung carcinoma. Osimertinib initiated after two to three month time point.

Initial slit-lamp examination was unremarkable. On detailed funduscopy of the left eye, a non-pigmented choroidal lesion was noted superior to the macula [Figure 1a] with subretinal fluid extending from the tumor into the subfoveal space [Figures 1b & 2a]. Fundus examination of the right eye was normal. Autofluorescence imaging of the left eye showed hyperfluorescence with a mixed or speckled pattern overlying the subretinal lesion [Figure 1b]. Optical coherence tomography (OCT) highlighted an abnormal foveal contour with subretinal fluid in the affected eye [Figure 2a]. B-scan ultrasound on the same visit demonstrated a 6.6 × 6.1 × 2.1 mm lesion in the left eye with heterogenous echogenicity [Figure 2b]. Screening computed tomography of the chest, abdomen, and pelvis highlighted a large 5.0 × 4.3 cm spiculated mass with central necrosis encasing the pulmonary vessels in the right upper lobe of the lung [Figure 3b]. In addition to the mass, enlarged lower paratracheal lymph nodes, lesions on the L4 spinous process, T1 vertebral body, and left adrenal nodule were noted and suspect of metastases.

Histological examination from fine needle aspiration of the lung mass and right lower paratracheal lymph node highlighted atypical cells and adenocarcinoma, respectively. Subsequent positive immunohistochemical staining with NapsinA, TTF- 1, BER-EP4, CK7, and Ki67 confirmed a malignancy of pulmonary origin. Positron emission tomography with Fluorodeoxyglucose (PET-FDG) revealed a right upper lobe pulmonary mass with extension into the right suprahilar region [Figure 3a], right paratracheal and mediastinal lymph node metastases, a 14-mm adrenal nodule, as well as T1 vertebral body, posterior iliac, and left acetabular bony metastases. MRI findings demonstrated a 4-mm enhancement in the left superior frontal gyrus suspect of brain metastases without significant mass effect. Due to the patient's “never- smoking” status, chances of finding a targetable driver mutation were high, and therefore, initiation of chemotherapy was withheld until sequencing was complete. Patient was then started on denosumab 120 mg every four weeks.

Two months after presentation, further ophthalmic work-up revealed gravitated subretinal fluid and decreased acuity of the affected eye (20/60). B-scan ultrasound highlighted an enlarged growth at 10.5 × 9.4 × 2.8 mm, with OCT showing worsening subfoveal fluid. Next-generation sequencing of the malignancy showed an EGFR pathogenic mutation variant. Patient subsequently began osimertinib 80 mg daily and denied targeted radiation therapy.

One month later, the patient's pin-hole acuity improved to 20/40, with color fundus photography showing an amelanotic choroidal lesion with subretinal fluid that had not grown, B-scan ultrasound showed a smaller mass (8.2 × 8.1 × 1.1 mm), and OCT revealed a flat lesion with decreasing subretinal fluid. A brain MRI highlighted resolution of the left frontal enhancement, while CT scans demonstrated interval decreases of lesions in the right upper lobe of the lung and some bony metastases, though Lesions in the T4 vertebral body and left acetabulum were unchanged.

Patient also exhibited a new 8-mm enhancement of the right hepatic lobe, not seen in earlier imaging. Intravenous chemotherapy was withheld as the interval change was markedly improved with osimertinib therapy alone.

Approximately nine months after diagnosis and daily treatment with oral osimertinib, patient was seen with resolving visual symptoms. Visual acuity of the left eye was correctable to 20/30. Fundus examination and B-scan ultrasound showed a grossly flat retina with no detectable mass and resolved subretinal fluid. OCT also demonstrated similar results. Most recent CT scan showed improved interval changes in the right lung mass and paratracheal lymph nodes, as well as a sclerotic appearance of the bony metastases and hypoattenuation of the previously noted liver enhancement.

##  DISCUSSION

Although ocular metastases are infrequent in lung cancer patients, ocular metastasis of pulmonary adenocarcinoma is less common, as adenocarcinoma spreads to the liver and adrenal glands more commonly.^[5]^ Additionally, the presence of choroidal metastasis may occur in up to 8.4% of primary cases of EGFR + NSCLC.^[6]^ The mean age of diagnosing uveal metastasis is 60 years and blurry or disturbed visual acuity is the most common presenting symptom.^[1,7,8]^ In addition to blurry vision, the patient did report mild fatigue and weight loss. Past studies have shown that choroidal metastasis (CM) has a yellow–white discoloration and is highly associated with subretinal fluid.^[1]^ Furthermore, the association of subretinal fluid and CM occurs in approximately 28.4% of patients.^[8]^ If these features are identified, it should initiate prompt investigation for a primary source. In the past, uveal metastasis in lung cancer generally carried a poor prognosis with mean time to death after discovery of 18.4 months in older patients (age 61–80).^[7]^ However, molecular profiling has recently led to improved treatment regimens, where osimertinib has shown increased median overall survival (38.6 months) compared to other EGFR-TKIs gefitinib and erlotinib (31.8 months) in treatment naïve NSCLC.^[9]^ Combinations of both systemic and external beam radiotherapies for CM previously showed lower regrowth rates,^[10,11]^ but therapies targeting driver mutations spare patients from ophthalmic adverse reactions to therapy, including radiation keratopathy, iris neovascularization and glaucoma, accelerated cataract formation, and optic neuropathy.^[12]^

Here, we present an informative depiction of the regression of ocular metastasis, where biopsy and next-generation sequencing directed the selection of osimertinib treatment due to its efficacy with EGFR-variant pulmonary adenocarcinoma and ability to penetrate the blood–brain barrier.^[3,4]^ This case adds to the growing body of evidence highlighting the utilization of molecular profiling to optimally treat choroidal metastasis of EGFR + NSCLC.^[13,14,15]^ However, in comparison to the some cases of ocular metastases treated with osimertinib, first- or second-generation TKIs were not attempted as first-line, as sequencing led to a more advantageous treatment plan that would spare adverse reactions to intravenous chemotherapy and targeted radiation.^[14,15]^ Additionally, we comprehensively correlate ophthalmic and systemic radiographic imaging over the time course of treatment to highlight visual symptomatic improvement alongside mass regression that was not described in earlier cases. However, our case also confirms prior experience in the field, where ophthalmic imaging offered a real-time, low-cost, and noninvasive monitoring of ocular metastasis and therapeutic response to newer agents like osimertinib.^[13,14,15]^

##  Ethics approval

This case report was conducted in accordance with the Declaration of Helsinki, data collection and evaluation were conducted in accordance with the Health Insurance Portability and Accountability Act (HIPAA).

##  Consent to Participate

Informed consent was obtained from all individual participants included in the study. Consent for publication was obtained according to the ICMJE Recommendations for Protection of Research Participants. The participant has consented to the submission of the case report to the journal.

##  Financial Support and Sponsorship

Nil.

##  Conflicts of Interest

There are no conflicts of interest.
